# Banxia Shumi decoction for the treatment of insomnia: A systematic review and meta-analysis

**DOI:** 10.1097/MD.0000000000046207

**Published:** 2025-11-28

**Authors:** Fang Wang, Lifang Wei

**Affiliations:** a Qingyunpu Campus, Jiangxi Psychiatric Hospital, Nanchang, Jiangxi Province, China.

**Keywords:** Banxia Shumi decoction, curative effect, insomnia, meta-analysis, systematic review

## Abstract

**Background:**

The efficacy of Banxia Shumi decoction (BSD) and its combination with Chinese medicine (acupuncture, formulas, etc) in treating insomnia remains controversial, with varying research conclusions. This study presents a systematic review and meta-analysis of its impact on insomnia.

**Methods:**

We conducted a comprehensive search of databases, including PubMed, Embase, Web of Science, and China National Knowledge Infrastructure. Studies meeting the criteria for the insomnia population, intervention, comparison, and outcome were independently screened and data extracted. Fixed- or random-effects models were used to calculate the standardized mean difference and 95% confidence interval (CI). Publication bias and research quality were evaluated to ensure reliability.

**Results:**

BSD was effective in treating method, as shown by improvements in clinical effective rate (risk ratio = 1.16, 95% CI: 1.10–1.23), Pittsburgh Sleep Quality Index (mean standard deviation [MD] = −3.22, 95% CI: −4.41 to − 2.03), Insomnia Severity Index (MD = −5.52, 95% CI: −6.29 to − 4.74), and Athens Insomnia Scale (MD = −1.62, 95% CI: −2.18 to − 1.06) were analyzed. According to the indicators, it has shown good effects in improving the symptoms of patients with insomnia. Compared to Western Medicine, the clinical efficacy of BSD + TCM (MD = 0.16, 95% CI: 0.08–0.24) was more significant than that of BSD alone. For primary insomnia, BSD was more effective (MD=−6.27, 95% CI: −6.43 to − 6.11, *P* < .0001).

**Conclusion:**

Based on the clinical efficiency index, BSD can effectively improve sleep and quality of life in patients with insomnia, shorten sleep onset time, increase sleep time and efficiency, and alleviate sleep and daytime dysfunction. However, the results should be considered carefully owing to some methodological weaknesses, including study diversity. Future research should employ standardized methods in large-scale, multicenter, randomized controlled trials to confirm these findings.

## 1. Introduction

Insomnia refers to one or more symptoms, such as difficulty falling asleep, early awakening, shallow sleep, and low-to-moderate sleep quality, occurring repeatedly despite adequate opportunities for sleep. It is a common sleep disorder that disrupts circadian rhythms and has been recognized as an independent disease in the 11th edition of the International Classification of Diseases-11.^[1]^ According to research by the World Health Organization, approximately 20% to 35% of the global population suffers from sleep disorders.^[2]^ The 2024 World Sleep Report states that the number of people with insomnia worldwide will exceed 2 billion. Long-term insomnia not only affects personal health and reduces quality of life,^[3]^ but can also lead to negative emotions such as anxiety and depression,^[4]^ exacerbating insomnia symptoms and creating a vicious cycle. Without timely and effective intervention, patients may experience chronic sleep disorders, daytime fatigue and weakness, decreased cardiovascular function, lack of concentration, anxiety, irritability, and other functional disorders that severely affect physical and mental well-being.^[5]^ Insomnia has gradually become a public issue, impacting national health, work productivity, family well-being, and social harmony, and has attracted social attention.^[6]^

From the perspective of Western medicine, insomnia is mainly associated with factors such as age differences, environmental and physiological changes, personal behavior, social psychology, neurological and mental disorders, medications, and diet. Internationally recognized conventional treatments for insomnia include cognitive-behavioral therapy and Western medicine; however, because of limited accessibility and side effects, these approaches do not meet the treatment needs of all patients.^[7]^ Currently, commonly used sedative-hypnotic drugs for treating insomnia include barbiturates and benzodiazepines. New agents mainly include non-benzodiazepines, melatonin receptor agonists, antihistamines, and appetite peptide receptor antagonists. While these drugs have a fast onset, significant therapeutic effects, and few “sleeping effects,” they are also associated with adverse reactions and the risk of drug dependence with long-term use.^[8]^

In traditional Chinese medicine (TCM), most practitioners believe that insomnia results from an imbalance between yin and yang.^[9]^ Similar interpretations are found in classical texts such as “*Huangdi Neijing·Ling Shu·Xieke.”*^[10]^ Herbs commonly used to treat insomnia include Banxia, Amber, Cinnabar, Magnet, Suanzaoren, Boziren, Yuanzhi, Hehuanpi, and Yejiaoteng, all of which are considered effective. Commonly used TCM prescriptions include Banxia Shumi decoction (BSD), Huanglian Ejiao decoction, Suanzaoren decoction, Guipi decoction, Wendan decoction, and Zhusha Anshen decoction. Modern pharmacological studies have shown that Banxia can inhibit the central nervous system and possesses analgesic and hypnotic effects.^[11]^ The Banxia decoction mentioned in *“Lingshu,”* also known as BSD, is one of the thirteen formulas in the *“Neijing”* and is specifically intended for treating insomnia. In TCM, conditioning therapies with minimal side effects and no risk of dependence are generally emphasized. Combining TCM and Western medicine can effectively improve the shortcomings of both.^[12]^ Furthermore, several randomized controlled trials (RCTs) suggest that BSD is effective for treating insomnia, especially in integrated TCM and Western medicine contexts. These studies have shown promising results, although challenges remain, such as changes in prescription and inappropriate usage.^[13–15]^ There is also room for further thinking, such as a systematic and comprehensive study on the efficacy of BSD to treat different symptoms of insomnia (e.g., primary insomnia or insomnia secondary to other conditions).

This study presents a meta-analysis on the efficacy of modified BSD, alone and in combination with other Chinese herbal medicines, in treating insomnia, aiming to provide a reference for clinical practice.

## 2. Materials and methods

This study has been registered on the International Platform of Registered Systematic Review and Meta-analysis Protocols (INPLASY) (Registration Number: INPLASY202590059; DOI: 10.37766/inplasy2025.9.0059, https://inplasy.com/inplasy-2025-9-0059/).

Ethics approval was not required, as this meta-analysis did not involve human or animal participants.

### 2.1. Search strategy

#### 2.1.1. Data retrieval

Literature was retrieved from databases including the China National Knowledge Infrastructure, Wanfang Database, VIP Journal Network, PubMed, Embase, and Web of Science. The search covered all articles from the establishment of each database up to June 2025. Chinese search keywords included “Banxia Shumi Tang,” “Integrated Traditional Chinese and Western Medicine,” “Insomnia Disorders,” and “Insomnia.” English search keywords included: Banxia Shumi Decoction, Insomnia, BSD, Integrated Chinese and Western medicine, Trial, and other related key terms. The search strategy used is presented in Table 1. Titles and contents of the included studies were checked manually to provide an objective summary evaluation and ensure that no relevant literature was omitted. A combination of systematic and intensive strategies was used to ensure broad coverage and a robust evidence base.

**Table 1:** Search strategy.

Search number	Query1 (CNKI, Web of Science, VIP Journal Network and Wanfang Database)	Query2 (PubMed, Embase)
#1	(Banxia Shumi decoction [Title/Abstract]) OR (integrated Chinese and Western medicine [Title/Abstract])	(Banxia Shumi decoction [Topic]) OR (BSD [Topic]) OR (integrated Chinese and Western medicine [Topic])
#2	(Insomnia [Title/Abstract]) OR (Insomnia Disorder[Title/Abstract])	(Insomnia [Topic]) OR (Insomnia Disorder [Topic])
#3	#1 AND #2	#1 AND #2

BSD = Banxia Shumi Decoction, CNKI = China National Knowledge Infrastructure.

#### 2.1.2. Inclusion criteria

Only RCTs published in English or Chinese were included, regardless of the publication journal. Eligible studies involved interventions using BSD alone or in combination with other drugs. Studies reported outcomes based on predetermined indicators, such as symptom improvement rate or reduction in insomnia severity. Ineffectiveness was defined as < 30% or one-third of the comprehensive feature score.

#### 2.1.3. Exclusion criteria

Studies were excluded if they did not meet the methodological standards required for systematic evaluation. Non-RCTs, duplicate publications, and studies with insufficient methodological quality were not considered. Animal studies, case reports, and review articles were also excluded to focus on clinical trials. Comparative studies lacking control groups were excluded. Additionally, articles in which ineffectiveness was not defined as a reduction of at least 30% or one-third of the comprehensive characteristics score, or were lacking extractable outcome indicators (including incidence rate or clinical efficacy) were excluded.

### 2.2. Quality evaluation criteria and data extraction

Two experienced researchers in this field independently screened the literature, extracted data, and assessed study quality using the Cochrane Risk of Bias 2 (RoB 2) tool. Discrepancies were resolved through discussion or by consulting a third reviewer. The extracted data included the study design, patient population, inclusion/exclusion criteria, intervention protocol, control treatments, and measured outcomes, with a particular focus on insomnia resolution scale scores. For missing data, authors were contacted when possible. If data could not be obtained, relevant outcomes were classified as secondary indicators, and subgroup analyses were performed accordingly.

### 2.3. Statistical methods

Revman 5.4 software was used for statistical analysis. For binary variables, relative risk (RR) was calculated with a 95% confidence interval (CI). For continuous variables, the mean standard deviation (MD) was reported with a 95% CI. Statistical heterogeneity was assessed using the *I*^2^ test. If *I*^2^ <50% and *P* > .05, heterogeneity was considered low, and a fixed-effects model was applied. If *I*^2^ >50%, indicating significant heterogeneity, a random-effects model was used for meta-analysis.^[16]^ When heterogeneity was too high for meta-analysis, a descriptive analysis was performed instead. Subgroup analyses were conducted when significant heterogeneity was detected and could potentially be explained by differences in study population characteristics. If the source of heterogeneity remained unclear, descriptive analysis was also performed.

### 2.4. Assessment of publication bias

Publication bias was assessed through funnel plot asymmetry and the Egger’s regression test. Potential biases in study selection and reporting were evaluated, and relevant explanations were provided during the meta-analysis process.

## 3. Results

### 3.1. Characteristics of included studies

**Figure 1.**
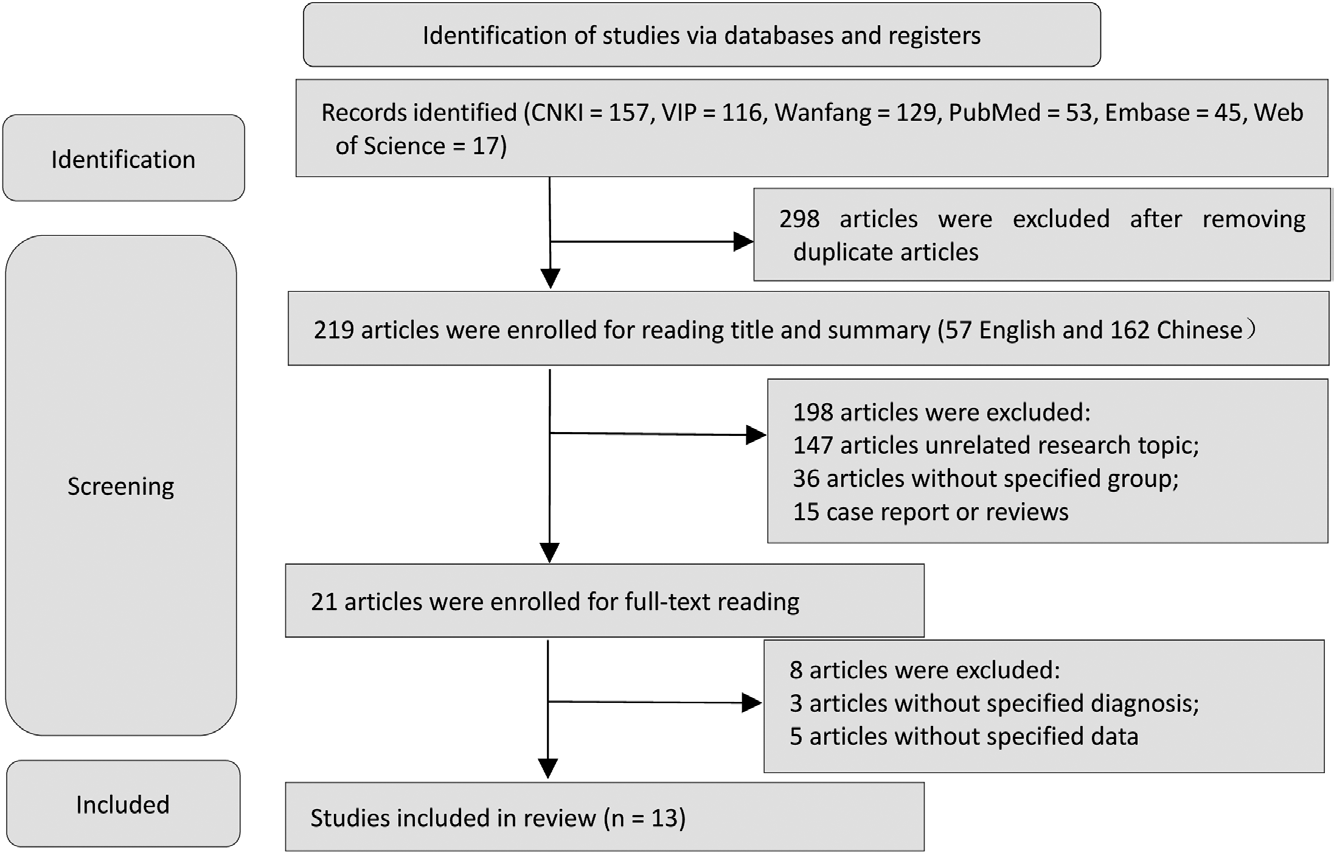
Literature selection framework. This is a flowchart depicting the study selection process, including the number of identified, screened, excluded, and included studies.

A total of 517 references were retrieved: 157 from China National Knowledge Infrastructure, 129 from the Wanfang Database, 116 from VIP, 53 from PubMed, 45 from Embase, and 17 from Web of Science. After removing duplicates, 219 references remained for screening using title and abstract. These were assessed based on the inclusion and exclusion criteria, resulting in 21 full-text articles reviewed for eligibility. Of these, 8 were excluded, and 13 were finally included. The screening process is illustrated in Figure 1. Baseline characteristics of the included studies are shown in Table 2.^[17–20,22–29]^

**Table 2:** Basic information of included studies.

Authors	Study type	Sample size	Gender (%F)	Age (mean [SD]	Course of disease (a, m)	Intervention	Dose (HM)/duration(days)	Outcomes
Wang^[17]^	RCT	70	44.29	T:40.61 ± 4.35 C:40.48 ± 4.33	T:3.11 ± 1.22 C:3.08 ± 1.16	T: BSD; C: Mianserin Hydrochloride	300ml, tid/42	a, b, c, i
Hao XM 2020^[18]^	RCT	100	53	T:55.3 ± 4.5 C:55.2 ± 4.3	NA	T: BSD; C: Estazolam	1dose, bid/28	a, d, e
Zhu^[19]^	RCT	60	36.67	T:47.48 ± 7.86 C:48.55 ± 7.56	NA	T: BSD; C: Zopiclone	1dose, bid/21	a, d, e
Li^[20]^	RCT	80	55	T:38.12 ± 5.28 C:38.66 ± 5.51	T:1.29 ± 0.29 C:1.26 ± 0.31	T: BSD + Eszopiclone; C: Eszopiclone	1dose, bid/28	a, e, f, g, i
Liang^[21]^	RCT	50	54	T:45.28 ± 2.36 C:42.18 ± 2.32	T:5.23 ± 0.28 C:5.22 ± 0.17	T: BSD + Acupuncture; C: Estazolam	1dose, bid/20	a, c, i
Chen^[22]^	RCT	46	56.52	T:41.66 ± 10.12 C:42.32 ± 9.44	T:1.56 ± 0.48 C:1.45 ± 0.51	T: BSD + TCM (WenDan Decoction); C: Estazolam	1dose, bid/30	h, i
Liu^[23]^	RCT	64	57.81	NA	NA	T: BSD + Acupuncture; C: Estazolam	1dose, bid/20	h, i
Xu^[24]^	RCT	60	38.33	T:55.55 ± 2.59 C:55.19 ± 2.58	T:3.43 ± 1.15 C:3.51 ± 1.12	T: BSD; C: Estazolam	1dose, bid/28	a, d, e
Pu^[25]^	RCT	80	0.45	T:42.61 ± 3.17 C:42.53 ± 3.14	T: 1.27 ± 0.46 C: 1.24 ± 0.36	T: BSD + TCM (WenDan Decoction); C: Estazolam	1dose, bid/30	h, i
Zhang^[26]^	RCT	52	48.08	NA	T: 5.48 ± 0.62 C: 5.50 ± 0.81	T: BSD + TCM (HuangLianWenDan Decoction); C: Alprazolam	1dose, bid/28	I
Guo^[27]^	RCT	60	48.33	T:42.33 ± 6.70 C:40.43 ± 8.60	NA	T: BSD; C: Eszopiclone	1dose, bid/21	a, i
Fu^[28]^	RCT	53	48.33	T:57.33 ± 5.33 C:55.08 ± 4.77	NA	T: BSD; C: Estazolam	1dose, bid/21	a, i
Song^[29]^	RCT	60	51.67	T:48.60 ± 8.57 C:44.83 ± 5.97	NA	T: BSD; C: ZaoRenAnShenKeLi	1dose, bid/21	a, h, i

(a) PSQI, (b) HAMD, (c) TESS, (d) SRSS, (e) ISI, (f) PSAS, (g) HAS, (h) AIS, (i) CEF, (j) SAS.

AIS = Athens insomnia scale, BSD = Banxia Shumi Decoction, CEF = clinical effective rate, HAMD = Hamilton Depression Scale, HAS = hyperarousal scale, ISI = insomnia severity index, PSAS = pre-sleep arousal scale, PSQI = Pittsburgh Sleep Quality Index, RCT = Randomized Controlled Trial, SAS = self-rating anxiety scale, SRSS = Self-rating Scale of Sleep, TESS = Treatment Emergent Symptom Scale.

### 3.2. Quality evaluation of included studies

**Figure 2.**
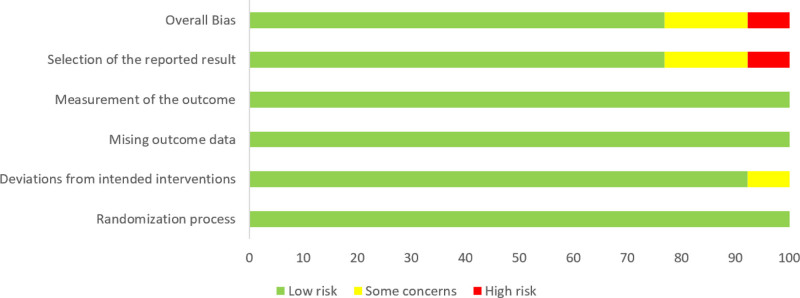
Quality evaluation of included studies. A risk of bias assessment chart displaying the methodological quality of the included studies based on Cochrane RoB 2. RoB = Risk of Bias.

**Figure 3.**
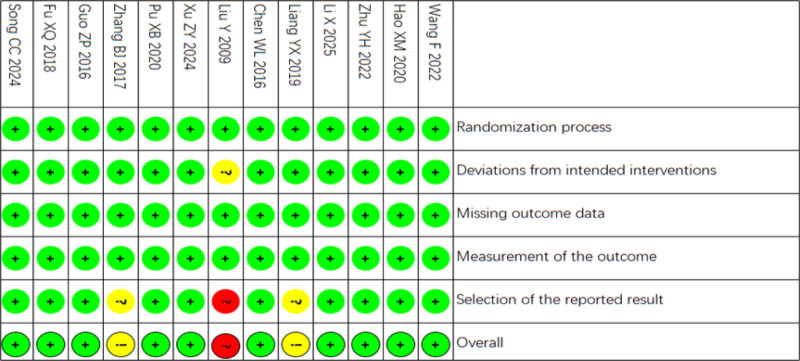
Risk of bias assessment of the included trials (traffic light plot).

Risk of bias (RoB) was assessed using the Cochrane RoB 2 Tool. Two independent reviewers performed the assessments. Any disagreements were resolved through discussion. The risk assessment of the literature quality is shown in Figures 2 and 3.

### 3.3. Results of meta-analysis

To comprehensively reflect the therapeutic effect of BSD on insomnia, the clinical effective rate (CEF) was used as the primary analytical indicator. The Pittsburgh Sleep Quality Index (PSQI), Insomnia Severity Index (ISI), and Athens Insomnia Scale (AIS) scales are commonly used to measure sleep level, with this study focusing primarily on PSQI scores. Given the characteristics of the included data, additional analyses were also conducted on ISI and AIS scores. Subgroup analysis to explore heterogeneity was mainly based on PSQI scores.

#### 3.3.1. Clinical effective rate

**Figure 4.**
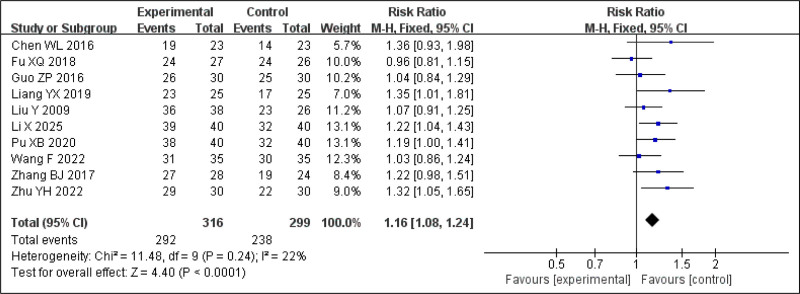
Forest plot of the meta-analysis for the total clinical response rate.

Ten RCTs reported clinical efficacy for BSD in the treatment of insomnia. There was no significant heterogeneity among these studies (*P* = .24, *I*^2^ = 22%). Therefore, a random-effects model was applied. The meta-analysis revealed a statistically significant difference in favor of the treatment group (RR = 1.16, 95% CI: 1.08–1.24, *P* < .0001), indicating that the CEF in the BSD group was 16% higher than in the control group (Fig. 4).

#### 3.3.2. PSQI scores

**Figure 5.**
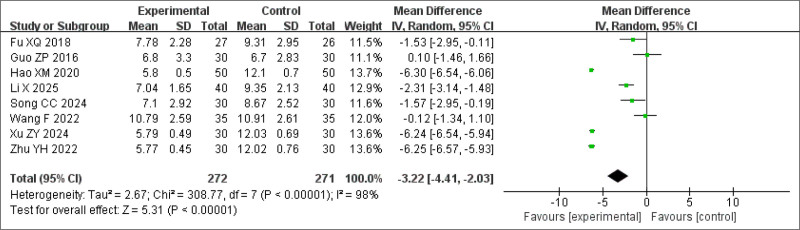
Forest plot of the meta-analysis for PSQI scores. PSQI = Pittsburgh Sleep Quality Index.

The PSQI score is a key indicator of sleep quality. Nine studies reported PSQI outcomes; however, in one study,^[27]^ only sub-item scores were reported, and the total PSQI score was not provided. Since the authors of this article were not contacted, and based on evaluator recommendations, this study was excluded from the analysis. The pooled analysis showed that the treatment group had a significantly greater reduction in PSQI scores than did the control group (MD = −3.22, 95% CI: −6.57 to −5.93, *P* < .0001) (Fig. 5). However, heterogeneity across studies was considerably high (*P* < .0001, *I*^2^ = 98%). Upon further examination, differences in insomnia subtypes, such as primary insomnia, refractory insomnia, and insomnia secondary to other conditions, were identified as potential sources of heterogeneity. Therefore, subgroup and sensitivity analyses were conducted, considering factors such as intervention type, sample size, treatment duration, and evaluation indicators.

#### 3.3.3. ISI scores

**Figure 6.**
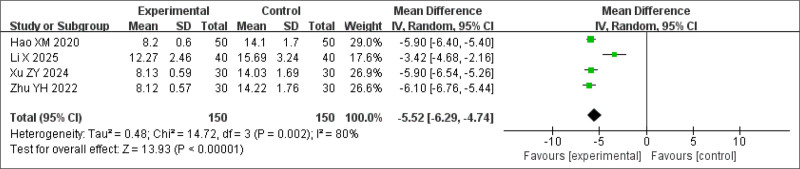
Forest plot of the meta-analysis for ISI scores. ISI = Insomnia Severity Index.

ISI scores were assessed in 4 studies. The analysis showed that the BSD treatment group demonstrated a significantly greater reduction in ISI scores than did the controls (MD = −5.52, 95% CI: −6.29 to −4.74, *P* < .0001) (Fig. 6). However, significant heterogeneity was observed (*P* < .0001, *I*^2^ = 80%).

**Figure 7.**
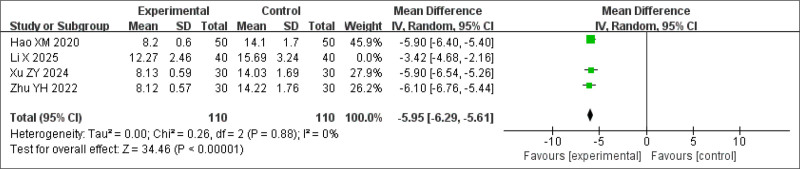
Sensitivity analysis of ISI scores. ISI = Insomnia Severity Index.

A sensitivity analysis was performed by excluding a study where BSD was used in combination with dexmedetomidine for treating insomnia because of phlegm-heat internal disturbance. After exclusion, heterogeneity was significantly reduced, while the treatment effect remained statistically significant (*P* < .0001, *I*^2^ = 0%) (Fig. 7).

#### 3.3.4. AIS scores

**Figure 8.**
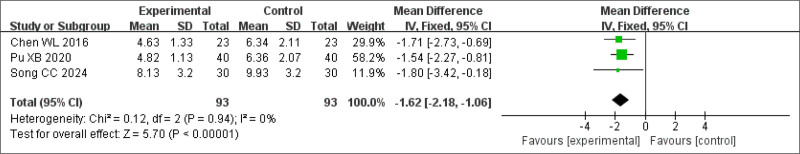
Forest plot of the meta-analysis for AIS scores. AIS = Athens Insomnia Scale.

AIS scores were evaluated in 3 articles. The meta-analysis showed that the treatment group experienced a significantly greater reduction in AIS scores than did the control group (MD = −1.62, 95% CI: −2.18 to −1.06, *P* < .0001) (Fig. 8). The difference between the 2 groups was statistically significant, and the combined effect size was reliable (*P* < .00001, *I*^2^ = 0%).

#### 3.3.5. Subgroup and sensitivity analyses

**Figure 9.**
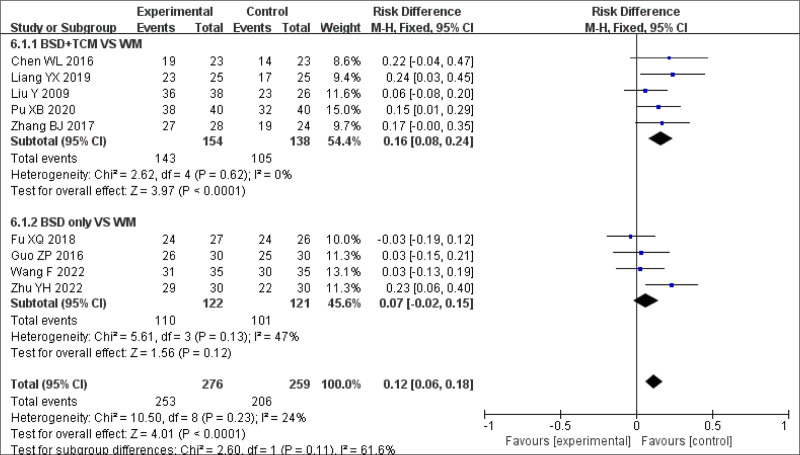
Subgroup analysis of clinical response rate.

**Figure 10.**
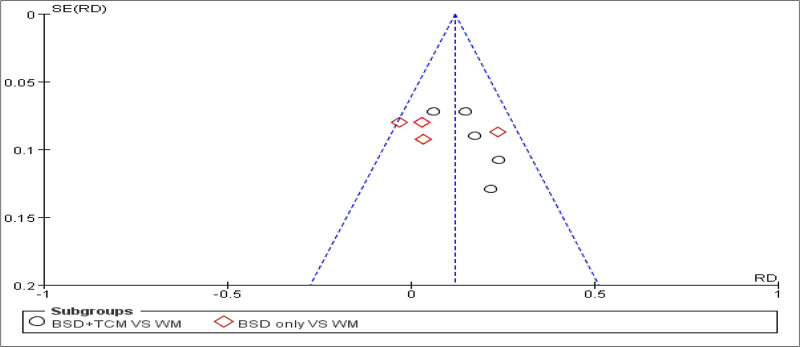
Funnel plot analysis of clinical response rate for subgroup analysis.

Among the 13 articles that met the inclusion criteria, only BSD was used as the intervention measure in 7 studies,^[17–19,29,22,26,20]^ BSD was combined with TCM (BSD + TCM) in 5 studies,^[27–29,23,25]^ while BSD was combined with Western medicine (BSD + WM) in 1 study.^[24]^ A subgroup analysis of clinical efficacy in the BSD + TCM group showed no significant heterogeneity between studies (*P* < .001, *I*^2^ = 0%), allowing for the use of a fixed-effects model. The combined analysis showed a statistically significant difference (MDBSD + TCM = 0.16, 95% CI: 0.08–0.24, *P* < .0001). The clinical efficacy of BSD + TCM was more significant than that of BSD only (Figs. 9 and 10).

**Figure 11.**
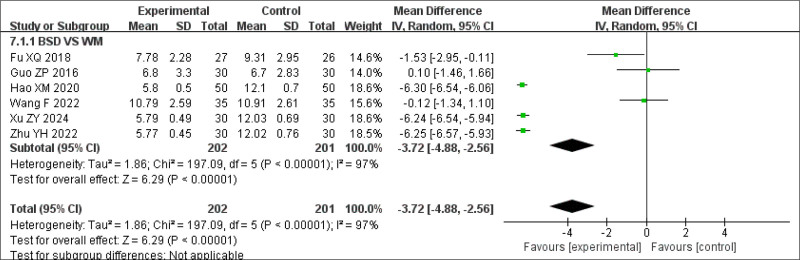
Subgroup analysis of PSQI scores. PSQI = Pittsburgh Sleep Quality Index.

Subgroup analysis of PSQI scores in the BSD group also showed statistically significant differences between the groups (MDBSD = −3.72, 95% CI: −4.88 to −2.56, *P* < .00001). Compared to Western medicine, the effect of BSD was significant (Fig. 11).

**Figure 12.**
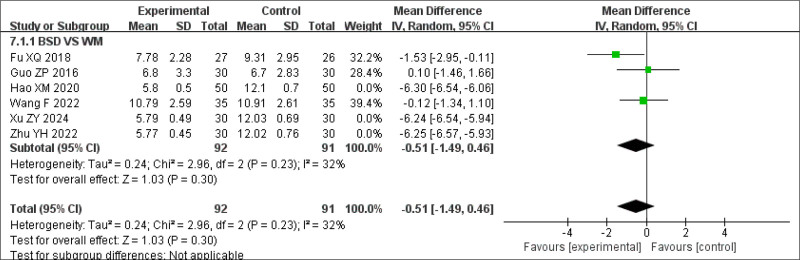
Sensitivity analysis of PSQI scores (excluding primary insomnia studies). PSQI = Pittsburgh Sleep Quality Index.

**Figure 13.**
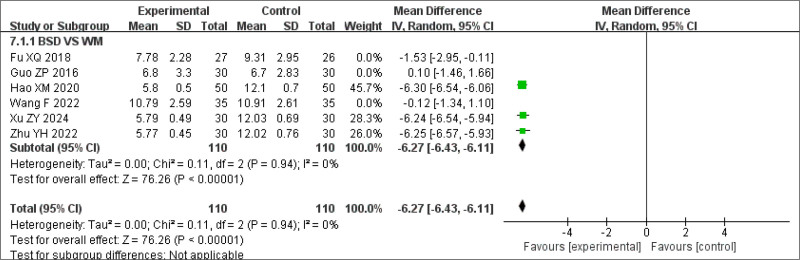
Sensitivity analysis of PSQI scores (excluding studies involving insomnia secondary to other diseases). PSQI = Pittsburgh Sleep Quality Index.

To explore sources of heterogeneity, a sensitivity analysis was conducted by excluding studies focused on primary insomnia. The remaining studies involved insomnia secondary to other conditions. The results were not statistically significant (Fig. 12). Another sensitivity analysis was performed by excluding 4 studies,^[17,29,26,20]^ each involving patients with comorbid conditions: depression with insomnia,^[17]^ postoperative insomnia after lung adenocarcinoma,^[20]^ other diseases accompanied by insomnia symptoms, such as damp heat disturbed insomnia,^[26]^ and BSD with Zaoren Anshen Keli.^[29]^ Owing to differences in symptoms, modifications to the BSD formulation were likely made to tailor treatment to specific conditions. The sensitivity analysis excluding these studies showed that the results remained statistically significant and consistent with the original analysis (MD = −6.27, 95% CI −6.43 to −6.11, *P* < .00001) (Fig. 13). This finding suggests that BSD is effective in treating insomnia symptoms, particularly in cases of primary insomnia. For patients with insomnia secondary to other conditions, individualized modifications of BSD may be necessary to achieve optimal clinical outcomes.

#### 3.3.6. Adverse effects rate

**Figure 14.**
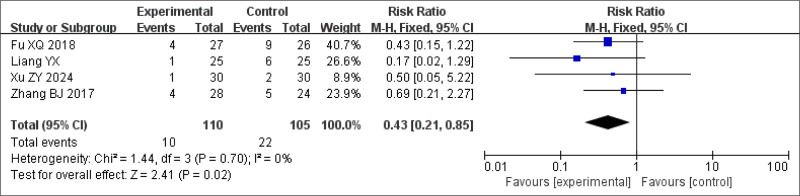
Forest plot of the meta-analysis for adverse event rates.

Adverse reaction rates associated with BSD treatment in patients with insomnia were reported in 4 studies.^[27,22,25,20]^ No significant heterogeneity was observed among these studies (*P* = .70, *I*^2^ = 0%), allowing the use of a fixed-effects model for meta-analysis. The results showed a statistically significant difference in favor of the BSD group (RR = 0.43, 95% CI: 0.21–0.85, *P* < .05), indicating a lower incidence of adverse reactions (including dizziness, headache, drowsiness, and fatigue) than that in the control group. This finding suggests that BSD had a better therapeutic effect (Fig. 14).

#### 3.3.7. Publication Bias

**Figure 15.**
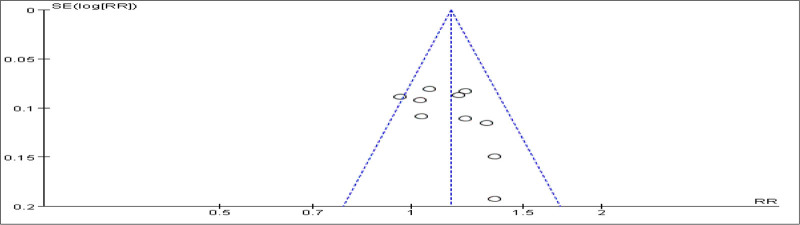
Funnel plot showing publication bias evaluation for clinical efficacy outcomes.

To evaluate publication bias, a funnel plot analysis was conducted based on the clinical efficacy results from 10 studies (Fig. 15). The distribution of points suggests potential publication bias, which may stem from factors such as non-publication of negative results and the inclusion of low-quality small-sample studies. To mitigate such bias in future studies, it is recommended to effectively use clinical trial registration platforms, include more “gray literature,” and reduce publication bias.

## 4. Discussion

Current treatment options for insomnia primarily include cognitive behavioral therapy for insomnia (CBT-I) and pharmacological interventions. However, CBT-I can be expensive, while Western medicine treatments often carry risks of drug dependence and substance abuse.^[30]^ BSD, a pure TCM formulation, offers a promising alternative. It is associated with fewer adverse effects, shows notable hypnotic efficacy, and provides additional benefits such as resolving phlegm, drying dampness, relieving nausea, and harmonizing the stomach.^[31,32]^ Evidence from this meta-analysis suggests that BSD is effective in improving insomnia symptoms, particularly in cases of primary insomnia, where results were more consistent and pronounced. For insomnia secondary to other diseases, modifications to the BSD prescription may be necessary based on individual patient symptoms.^[33]^ In some studies, BSD effectively replaced Western medications such as zopiclone, estazolam, and mianserin without causing severe discomfort, suggesting good tolerability.

However, this study has some limitations. High heterogeneity was observed across the included studies. Although sensitivity and subgroup analyses were conducted, some outcome measures still exhibited heterogeneity, which may increase the risk of potential bias. In terms of the clinical efficacy of the PSQI,^[27]^ studies lacked a unified clinical efficacy evaluation standard, which, to some extent, reduces the strength and credibility of the research evidence. Differences in application, prescription, medication, course of treatment, sample size, and published data among the included studies may have reduced the reliability of the results of this meta-analysis. In some studies, there was no long-term follow-up, and endpoint events were often not reported. A publication bias exists in the included literature; the funnel plot of clinical efficacy shows asymmetry, and there may be some selective reports.

## 5. Conclusion

This systematic review and meta-analysis suggest that BSD may improve sleep quality and some outcomes in patients with insomnia, such as sleep onset latency, total sleep time, and sleep and daytime functioning. However, the findings should be interpreted cautiously because of heterogeneity among studies and the generally low quality of evidence. Further well-designed, multicenter RCTs with standardized protocols are needed to more definitely assess the efficacy and safety of BSD for insomnia treatment.

## Author contributions

**Conceptualization:** Fang Wang, Lifang Wei.

**Data curation:** Fang Wang.

**Formal analysis:** Fang Wang, Lifang Wei.

**Funding acquisition:** Fang Wang.

**Methodology:** Fang Wang, Lifang Wei.

**Project administration:** Fang Wang.

**Resources:** Fang Wang.

**Software:** Fang Wang.

**Writing – original draft:** Fang Wang, Lifang Wei.

**Writing – review & editing:** Fang Wang, Lifang Wei.
